# Electron Heat Source Driven Heat Transport in GaN at Nanoscale: Electron–Phonon Monte Carlo Simulations and a Two Temperature Model

**DOI:** 10.3390/ma15051651

**Published:** 2022-02-23

**Authors:** Anish Muthukunnil Joseph, Bingyang Cao

**Affiliations:** Key Laboratory of Thermal Science and Power Engineering, Ministry of Education, Department of Engineering Mechanics, Tsinghua University, Beijing 100084, China; a-ls18@mails.tsinghua.edu.cn

**Keywords:** electron–phonon interaction, electron–phonon Monte Carlo, two temperature model, Boltzmann transport equation

## Abstract

The thermal energy transport in semiconductors is mostly determined by phonon transport. However in polar semiconductors like GaN electronic contribution to the thermal transport is non-negligible. In this paper, we use an electron–phonon Monte Carlo (MC) method to study temperature distribution and thermal properties in a two-dimensional GaN computational domain with a localized, steady and continuous electron heat source at one end. Overall, the domain mimics the two-dimensional electron gas (2DEG) channel of a typical GaN high electron mobility transistor (HEMT). High energy electrons entering the domain from the source interact with the phonons, and drift under the influence of an external electric field. Cases of the electric field being uniform and non-uniform are investigated separately. A two step/temperature analytical model is proposed to describe the electron as well as phonon temperature profiles and solved using the finite difference method (FDM). The FDM results are compared with the MC results and found to be in good agreement.

## 1. Introduction

Electrons and phonons are the thermal energy carriers in solids in general [1]. While electrons have a dominant contribution to the thermal conductivity in metals, phonons play the major role in semiconductors and insulators. Over the past few decades, rapid advancements in first-principle simulation and thermal metrology have led to an elaborate understanding of the thermal transport properties of electrons and phonons. However, electrons’ significance in thermal transport in semiconductors started to be appreciated only recently [2]. Initially, the efforts were merely focused on the influence of electron–phonon interaction (EPI) on the properties of electrons in metals and semiconductors, including the explanations for the temperature dependence of electrical conductivity and electronic thermal conductivity. Very much less attention was given to the lattice thermal conductivity, or the thermal conductivity governed by phonons, since in metals, it is generally believed that phonon contribution to thermal conductivity is negligible compared to the electronic counterpart. On the contrary, thermal conduction is dominated by phonons in semiconductors; the impact of EPI on phonon transport receives less attention due to relatively low carrier concentrations which limits the scattering of phonons by electrons much less important than the phonon–phonon scattering. The first study in this regard was done by Sommerfeld and Bethe [3]; they calculated the relaxation time of phonons incorporating EPI in metals. Based on that, Makinson [4] proposed an expression for lattice thermal conductivity of metals as a function of temperature, where he concluded that the electrons interact equally with longitudinal and transverse phonons, different from Bloch’s coupling scheme [5] that restricts the electrons so that they only interact with longitudinal phonon modes. A work by Jia-Yue Yang et al. studied the Frolich EPI contribution to the thermal conductivity of GaN. They found that the lattice thermal conductivity of GaN is decreased by 24–34% [6] after incorporating EPI.

Among the analytical and numerical methods available to solve the Boltzmann transport equation (BTE), Monte Carlo methods are found to be dominant in terms of flexibility, efficiency and accuracy. The phonon tracing and tracking strategy used in this work are found in literature like the MC study on phonon diffusive-ballistic heat conduction in silicon nanofilms by Y. C. Hua, B. Y. Cao [7] and many more. MC simulations in general can be slow and computationally expensive depending on the amount of physics involved. However, they are found to be very successful in predicting the thermal conductivities of nano-structures. Having said that, there is still a plenty of room for improvements in the algorithm such as electron–phonon coupling and its impacts on thermal transport, ballistic and Fourier regime studies on the external/internal heat source driven thermal transport, etc.

The present work involves an MC study on a two dimensional GaN channel similar to that found in GaN-based HEMTs. The presence of a non-zero gate voltage is responsible for the nominal device operation and volumetric heat generation (hot spot) at the drain side edge of the gate due to accumulated charge distribution. Electron source induced thermal transport and development of an analytic model to describe it effectively are the main scope of this work. Some recent works on the electrothermal properties simulation and modeling on GaN HEMTs are the following: Mei wu et al. [8] proposed an electric method for the estimation of temperature in the AlGaN/GaN HEMT channel and also they built a 2D electrothermal model to describe the findings; Bikramjith chatterjee et al. [9] examined the self heating effects on HEMTs using UV thermoreflectance imaging; Luoyun Yang et al. [10] studied the electrothermal mechanism of GaN HEMT and proposed a two-dimensional analytic model for the device; Yu-Chao Hua et al. [11] investigated ballistic-diffusive regime thermal spreading resistance in GaN HEMTs; and Qing Hao et al. [12] used a coupled electron–phonon MC to investigate temperature distribution in GaN HEMT more accurately. Drawing motivation from the past studies on electrothermal properties of HEMT channel like nano-structures, we introduce an electron–phonon Monte Carlo study on a GaN computational domain with a localized, steady electron heat source. High energy electrons entering continuously from source terminal to the channel drift under the influence of the electric field. The external electric field ($\vec{F}$) acts as a volumetric, indirect internal heat source by pumping energy to the electrons. As they travel across, electrons interact with lattice vibrations/phonons, thereby exchanging energy and momentum with them. This exchange of energy and momentum is the essence of electron–phonon MC simulation. The electron–phonon interactions (EPIs) are incorporated by accounting for various modes of electron–phonon scattering mechanisms such as acoustic phonon scattering (LA), optical phonon scattering (LO), polar optical phonon scattering (POP), and inter-valley scattering (IV). The EPI is dominated by the emission of a large number of energetic phonons into the computational domain by the electrons. They are tracked along with the electrons to get an ensemble of trajectories that can give an elaborate picture of the electrothermal properties of the material.

The two temperature models (TTM) are generally formulated within the space–time continuum using differential calculus. In the past few decades, there has been significant development in discrete approaches where, on the contrary, it is assumed that space and time are discrete variables. In those models both space and time are discretized. At the same time the dynamic variables are permitted to take a continuum of values. The discrete model provides heat and mass transport differential equations in discrete form, so it can be in particular suitable for numerical methods like FEM and FDM of cellular systems. We have derived a two step model from a conventional parabolic two step/temperature model that has been used for a while to deal with ultra-fast heating phenomena such as pulsed laser, electron beam induced heating, etc. There have been a number of works based on the two temperature model in the past few decades [13,14,15,16], particularly with rapid advancement in the ultra-fast laser technologies. Niu and W. Dai [17] developed an implicit finite difference scheme on a grid based on the two temperature hyperbolic model (TTHM) for thermal deformation in a two-dimensional double layered thin film irradiated by ultrashort laser pulses. Their study accounted for the coupling effect between strain rate and lattice temperature, as well as for the hot electron blast effect in momentum transfer. Roth et al. [18] coupled the molecular dynamic method with the TTHM. They demonstrated that, for copper, a difference between predictions of the local equilibrium TTM and the TTHM of about 1000 K in the maximum electron temperature and about 80 K in the final lattice temperature has been observed. Our MC simulation is in fact aimed to verify the accuracy of the newly developed parabolic two step model for dealing with localized electron source driven thermal transport under the external electric field.

## 2. Electron–Phonon Monte Carlo Method

In the MC simulation technique, point particles (such as electrons, phonons, holes, etc.) are drawn, distributed in the computational domain and let evolve in time. Individual trajectories of the particles are tracked by imposing various scattering mechanisms.

### 2.1. Phonon Monte Carlo

The phonon tracing MC algorithm [7] is used in this work where phonon BTE is solved under the relaxation time approximation, thereby computational particles describe only deviation from the equilibrium distribution. The phonon tracing MC cuts down the computational time significantly in comparison with the phonon ensemble MC [19]. In this study, phonon dispersion relations are calculated using the “Brillouin zone boundary condition” (BZBC) model proposed by Chung et al. [20]. The relaxation time formulations by Holland [21] are also used. The energy of each phonon bundle is directly related to the frequency by E(ω)∝ℏω. Hence, initializing the frequency would, by default, set the energy too. The polarization and frequency of the phonon bundles are assigned using the schemes provided in references [22,23], respectively. The role of optical phonons in the heat transport is negligible as their group velocities are very low compared to longitudinal acoustic (LA) and transverse acoustic (TA) modes. However, they play a very active and dominant role in interacting with electrons, thereby causing an indirect impact on the temperature distribution and other thermophysical properties. We know that longitudinal optical (LO) phonons in wurtzite GaN eventually decay into a large wave vector transverse optical (TO) and LA/TA phonon branches [24,25] i.e., LO⟶ TO + LA or LO⟶ TO + TA; the resulting LA/TA phonons are then tracked successfully. The typical lifetime of LO phonons in GaN is about 3–4 ps at room temperature [24].

Some aspects of a modified phonon MC scheme in Ref. [26] are used in this work. The key feature of the scheme is that a “reference temperature” ($T_{ref}$) is defined to cut down the computational expenses and statistical noises significantly. The “reference temperature” is usually fixed to be minimum temperature in the domain (in our case, 300 K, the room temperature). Phonon bundles emitted by the electron bundles are tracked by taking all scattering events into account. The phonons are moved from one position to the another ballistically in the time interval $▵t_{p}$; either 3-phonon or Umpklapp or impurity scattering are chosen probabilistically. Those phonons encountered with constant temperature boundaries are absorbed and with adiabatic boundaries are reflected either specularly or diffusively. Local temperature is calculated at the end of each drift. In order to calculate the local/pseudo temperature, it is necessarily assumed that the total energy carried by the phonon bundles in the local spacial element is equal to the energy calculated using the Bose–Einstein distribution for the same element. Therefore, the pseudo-temperature is given by

(1) $$\sum_{p}\sum_{i=1}^{N_{b}}\hbar \omega_{i}\left[N\left(\omega_{i},T_{pseudo}\right)-N\left(\omega_{i},T_{ref}\right)\right]D_{i}\left(\omega_{i},p\right)▵\omega_{i}=\frac{E(x,y,z)}{▵x▵y▵z},$$

where E(x,y,z) is the net energy carried by the phonon bundle and *N* is the Bose–Einstein function. The effective phonon relaxation time $\tau_{p}$ is written as

(2) $$\frac{1}{\tau_{p}}=\frac{1}{\tau_{I}}+\frac{1}{\tau_{U}}+\frac{1}{\tau_{N}}.$$

The probability of a phonon undergoing scattering between time *t* and t+▵t is given by,

(3) $$R_{scat}=1-\exp\left(\frac{-▵t}{\tau_{p}}\right).$$

Deciding which scattering is to be undergone is done as follows: First, the probability of impurity scattering (β) is calculated using [23],

(4) $$\beta =\frac{\tau_{I}^{-1}}{\tau_{I}^{-1}+\tau_{3ph}^{-1}}.$$

Next a random number *r* is drawn and compared with β. If r<β, there is impurity scattering; otherwise three phonon scattering occurs. Impurity scattering is implemented by assigning a new random direction to the incident phonon, assuming isotropic impurity distribution. Otherwise, for three phonon scattering, the phonon is absorbed, the track of its path is terminated and a new phonon drawn from the equilibrium distribution at $T_{pseudo}$ is emitted and tracked.

### 2.2. Electron Monte Carlo

This is a semi-classical MC approach of simulating carrier transport in semiconductors [27]. Assuming the carrier motion comprised of free flights encountered by various scattering mechanisms, a computer may be often used to simulate the trajectories of particles (electrons, holes, etc.), as they move across the device under the influence of an external electric field, applying classical mechanics. The scattering events and the duration of particle drift are obtained using random numbers. The method is equivalent to solving BTE for electrons and takes the form,

(5) $$\frac{\partial f}{\partial t}+\frac{1}{\hbar }{▽}_{k}E\left(k\right){▽}_{r}f+\frac{eF(r)}{\hbar }{▽}_{k}f={\left[\frac{\partial f}{\partial t}\right]}_{coll},$$

where *f* is the electron distribution, *E* is the energy, *e* is the electron charge and F(r) is the external field. Parabolic energy bands are assumed for this work. In the case of GaN, E(k) takes the form,

(6) $$E\left(k\right)=\frac{\hbar^{2}}{2}\left[\frac{k_{l}^{2}}{m_{l}^{*}}+\frac{2k_{t}^{*}}{m_{t}^{2}}\right],$$

where $m_{l}^{*}$ and $m_{t}^{*}$ are longitudinal and transverse effective masses, respectively (for GaN $m_{l}=0.2m_{0}$, $m_{t}=0.2m_{0}$).

#### Scattering Mechanisms

A computationally efficient approach to including scattering in MC is to store and use individual scattering rates obtained using Born approximation. The Fermi golden rule gives the first order transition probability per unit time for a scattering from a state $|k\rangle$ to a state $|k^{\prime }\rangle$ and can be found in Ref. [27]. In order to get a complete understanding of the scattering processes, one has to consider all such scattering rates $\lambda_{1},\lambda_{2},\lambda_{3}\ldots ,\lambda_{n}$, then calculate the total scattering rate given by,

(7) $$\lambda_{tot}=\sum_{i=1}^{n}\lambda_{i}.$$

The calculation of the phonon scattering rate for LA using the Fermi golden rule and rigorous algebra takes the form [27],

(8) $$\lambda_{LA}=\frac{\sqrt{2}}{\pi }\frac{Z_{A}^{2}\sqrt{m_{l}^{*}m_{t}^{*2}}k_{b}T}{\rho \hbar^{4}v_{s}^{2}}\sqrt{E}.$$

Similarly, for optical phonon deformation potential interaction, the longitudinal optical phonon scattering (LO) rate is derived as [27],

(9) $$\lambda_{LO}=\frac{D^{2}\sqrt{m_{l}^{*}m_{t}^{*2}}}{\sqrt{2}\pi \hbar^{3}\rho \omega_{o}}\left[N_{q}\sqrt{E+\hbar \omega_{0}}+\left(N_{q}+1\right)\sqrt{E-\hbar \omega_{o}}\right].$$

In order to calculate adsorption and emission probabilities, we define $\lambda_{LO}=\frac{1}{\tau_{LO}^{opd}}$. It is crucial to break Equation (9) into two parts: Absorption and emission rates,

(10) $$\lambda_{aLO}=\frac{D^{2}\sqrt{m_{l}^{*}m_{t}^{*2}}}{\sqrt{2}\pi \hbar^{3}\rho \omega_{o}}\left[N_{q}\sqrt{E+\hbar \omega_{0}}\right].$$

(11) $$\lambda_{eLO}=\frac{D^{2}\sqrt{m_{l}^{*}m_{t}^{*2}}}{\sqrt{2}\pi \hbar^{3}\rho \omega_{o}}\left[\left(N_{q}+1\right)\sqrt{E-\hbar \omega_{o}}\right].$$

Now, the probabilities that an electron will undergo absorption and emission are given by,

(12) $$P_{a}=\frac{\lambda_{aLO}}{\left(\lambda_{aLO}+\lambda_{eLO}\right)},$$

and

(13) $$P_{e}=\frac{\lambda_{eLO}}{\left(\lambda_{aLO}+\lambda_{eLO}\right)},$$

respectively. It is noted that $P_{a}+P_{e}=1$. A random number *r* is drawn from [0 1]. If $r<P_{a}$, there is absorption; otherwise, emission occurs.

Electrons can also be scattered by polar optical phonons. This mechanism is dominant in GaN and it is also called polaron scattering. Polar optical phonon scattering (POP) arises from the polarities of the two different atoms in the compound. The total POP scattering rate derived using the Fermi golden rule is given in a straight forward manner by [28],

(14) $$\lambda_{POP}=\frac{e^{2}\omega_{o}(\frac{K_{0}}{K_{\infty }}-1)}{2\pi K_{0}\epsilon_{0}\hbar \sqrt{2\frac{E}{m^{*}}}}\left[N_{q}\sinh^{-1}\sqrt{\frac{E}{\hbar \omega_{o}}}+\left(N_{q}+1\right)\sinh^{-1}\sqrt{\frac{E}{\hbar \omega_{o}}-1}\right],$$

where $K_{0}$ is the static dielectric constant, $K_{\infty }$ is the high frequency dielectric constant, $\epsilon_{0}$ is the permittivity of free space, $N_{q}$ is the Bose–Einstein function, *ℏ* is Planck’s constant, *E* is the electron energy. Absorption and emission rates are obtained separately as,

(15) $$\lambda_{aPOP}=\frac{e^{2}\omega_{o}\left(\frac{K_{0}}{K_{\infty }}-1\right)}{2\pi K_{0}\epsilon_{0}\hbar \sqrt{2\frac{E}{m^{*}}}}\left[N_{q}\sinh^{-1}\sqrt{\frac{E}{\hbar \omega_{o}}}\right],$$

(16) $$\lambda_{ePOP}=\frac{e^{2}\omega_{o}\left(\frac{K_{0}}{K_{\infty }}-1\right)}{2\pi K_{0}\epsilon_{0}\hbar \sqrt{2\frac{E}{m^{*}}}}\left[\left(N_{q}+1\right)\sinh^{-1}\sqrt{\frac{E}{\hbar \omega_{o}}-1}\right],$$

respectively.

Probabilities of emission and absorption are given by,

(17) $$P_{a}=\frac{\lambda_{aPOP}}{\left(\lambda_{aPOP}+\lambda_{ePOP}\right)},$$

(18) $$P_{e}=\frac{\lambda_{ePOP}}{\left(\lambda_{aPOP}+\lambda_{ePOP}\right)}.$$

Intervalley scattering (IV) takes place when electrons are scattered between different valleys. Generally speaking, there is a significant wave vector change for electrons to transit between valleys, and therefore an optical phonon is often needed to support the scattering process. Intervalley optical phonon scattering is very significant for high-energy electrons like those are found in GaN under strong electric fields. Concerning $D_{ij}$, the intervalley deformation potential is used for calculating the scattering rate of an electron from its initial *i*th valley into the final *j*th valley. The corresponding absorption and emission rates are given by,

(19) $$\lambda_{aIV}=\frac{D_{ij}^{2}Z_{ij}\sqrt{m_{l}^{*}m_{t}^{*2}}}{\sqrt{2}\pi \hbar^{3}\rho \omega_{o}}\left[N_{q}\sqrt{E+\hbar \omega_{0}-▵E_{ij}}\right],$$

(20) $$\lambda_{eIV}=\frac{D_{ij}^{2}Z_{ij}\sqrt{m_{l}^{*}m_{t}^{*2}}}{\sqrt{2}\pi \hbar^{3}\rho \omega_{o}}\left[\left(N_{q}+1\right)\sqrt{E-\hbar \omega_{o}-▵E_{ij}}\right],$$

where $Z_{ij}$ is the number of equivalent intervalley branches and is equal to the product of the numbers of equivalent valleys for the *i*th and *j*th valleys. $▵E_{ij}$ is the difference in energy between the bottoms of the *j*th and *i*th valleys. The total intervalley scattering is obtained by

(21) $$\lambda_{IV}=\lambda_{aIV}+\lambda_{eIV}.$$

Ionic impurity (IM) scattering is another form of scattering encountered by the electrons which becomes extremely elastic in nature. For an ionized impurity, the scattering source can be characterized by screened Coulomb potential. Typically, the ionic impurity density varies between $10^{15}$ and $10^{17}$ (cm${}^{-3}$). Time dependent perturbation analysis yields a net scattering rate of [28],

(22) $$\lambda_{I}=\frac{N_{I}e^{4}}{16\sqrt{2m^{*}}\pi K_{0}^{2}\epsilon_{0}^{2}}\left[\ln\left(1+\gamma^{2}\right)-\frac{\gamma^{2}}{1+\gamma^{2}}\right]E^{-3/2},$$

where $N_{I}$ is the impurity density.

(23) $$\gamma =\frac{8m^{*}EL_{D}^{2}}{\hbar^{2}},$$

where $L_{D}$ is the Debye length, given by,

(24) $$L_{D}=\sqrt{\frac{K_{0}\epsilon_{0}k_{b}T}{e^{2}n_{e}}},$$

where $n_{e}$ is the electron number density, $\frac{1}{\tau_{eI}}=\lambda_{I}$. The effective relaxation time can be calculated using Mathissen’s rule as,

(25) $$\frac{1}{\tau_{e}}=\frac{1}{\tau_{eI}}+\frac{1}{\tau_{POP}}+\frac{1}{\tau_{LO}}+\frac{1}{\tau_{LA}}+\frac{1}{\tau_{IV}}.$$

The mean free path of electrons is calculated using,

(26) $$l_{e}=v_{th}\tau_{e},$$

where $v_{th}$ is the average thermal speed of electrons which is of the order $10^{5}$ (m/s). Based on the relaxation time approximation, the traveling distance of electrons can be obtained by,

(27) $$\vec{r_{e}}=\vec{r_{e0}}+▵t_{e}\vec{v_{e}},$$

where $\vec{v_{e}}$, $\vec{r_{e}}$ and $\vec{r_{e0}}$ are the instantaneous electron velocity and initial and final position vectors, respectively.

Scattering rates for 3D GaN material are used for 2D channel for simplicity. It has been seen that 3D material treatment for polar optical phonon scattering is justifiable for 2DEG as well [12]. Since the electron energies are high, the only dominant players of scattering are polar optical phonon and intervalley; both in 3D are used for the 2D domain also [12]. We state that approximating 3D bulk scattering for simulating electron transport in the 2D channel is a clear limitation of our approach which might have caused compromises concerning the accuracy of the electron part of the MC simulation. The mobility model in M. Shur et al. [29] is used in this work where they have derived electron mobility models induced by various scattering modes addressed separately for 2DEG and bulk scenarios. Some other relevant works dealing with various scattering and associated electron mobilities of 2DEG in GaN based HEMTs can be found in I. Berdalovic et al. [30,31], Bag et al. [32], Y. J. Chai et al. [33] and T Fang et al [34]. Another notable work worth mentioning in this context is J. Zhang et al. [35] on the mobility of 2DEG in AlGaN/GaN heterostructures with varying Al content.

## 3. Numerical Scheme

**Figure 1:**
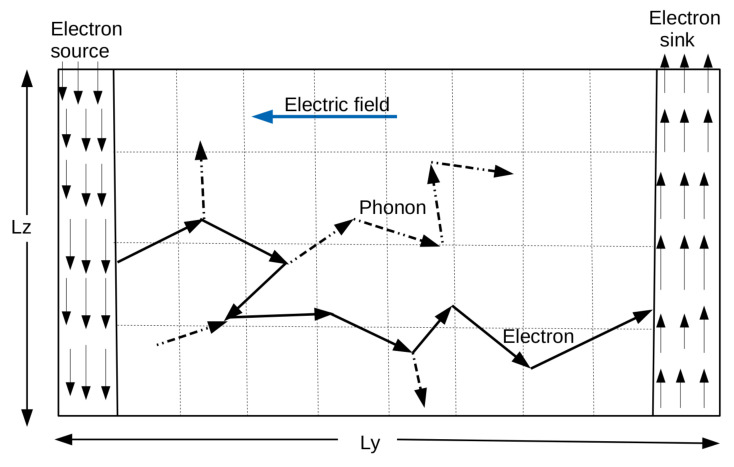
Front/source and rear/sink boundaries are set to be isothermal at room temperature. Lateral boundaries are shown to be adiabatic.

In order to explore electron–phonon coupled transport at nano-scales, we implement electron–phonon MC within a two dimensional rectangular geometry of size $L_{y}\times L_{z}$ as seen in Figure 1. The electron source and sink are located at the rear and front ends. Front and rear boundaries are set to be isothermal at room temperature where high energy electrons enter and the leave the domain, respectively. The whole system is discretized into a number of rectangular control elements each of size ▵y×▵z; these are shown in Figure 1 using dotted lines. Lateral boundaries are adiabatic and therefore, specularly or diffusively reflective. The time steps $▵t_{p}$ and $▵t_{e}$ are crucial in deciding the overall thermal and electron transports.

If $▵t_{p}$ is too large the scattering probability will be always 1, providing unrealistic results, the same is true with $▵t_{e}$ also. At the same time, time steps should be carefully selected such that the ballistic distance of the fastest phonon/electron does not exceed the smallest space step (i.e., ▵y and ▵z). The parameters of GaN used in the computation are acoustic deformation potential $Z_{A}$ = 8.3 eV, the number of equivalent intervalley routes $Z_{if}=3$, intervalley deformation potential $D_{if}=1.0\times 10^{11}$ eV/m, optical deformation potential $Z_{o}$ = 4.5 eV, and LO phonon frequency $\omega_{o}$ = 20 THz [6,36,37].

A detailed step by step algorithm of the phonon–electron MC that describes the entire process is given by:

1. First, $N_{e}$ the total number of electron bundles per unit time, np the phonon count and ne the electron count are initialized. Then ne is incremented as the the first electron bundle leaves the source. For each electron bundle, an initial energy, random $\vec{k}$ vector, random position and direction are assigned.
2. Once an electron bundle is emitted from the source, its count, ne, is incremented by one. At the end of each drift, the $\vec{k}$ vector, energy, velocity and the position of the electron bundle are updated as follows: $\vec{k_{f}}=\vec{k_{i}}-\frac{e\vec{F}▵t_{e}}{\hbar }$, $E=\frac{\hbar^{2}k_{f}^{2}}{2m^{*}}$, $\vec{v_{e}}=\frac{\hbar \vec{k_{f}}}{m^{*}}$, $\vec{r_{f}}=\vec{r_{i}}+\vec{v_{e}}▵t_{e}$.
3. Electron scattering is chosen probabilistically among LA, LO, IM, IV and POP. In the case of LA scattering, the LA phonon count is updated in the corresponding spacial bin. POP, IV and LO counts are updated by subtracting or adding phonons, depending on whether absorption or emission takes place (at the same time the electron energy is further modified as $E_{f}=E_{i}\pm \hbar \omega$). IV is found to be negligible except for higher energies. In the case of emission, the emitted phonon is tracked until it gets absorbed by either of the isothermal boundaries and the control is then returned back to the electron that had emitted the phonon and continues with its tracking. The energy of the electron is then reduced by that of the emitted phonon and the same is added to the corresponding spacial bin, thereby accounting for the energy conservation. If the emitted phonon is of the LO branch whose group velocity is marginal, control waits for it to decay into LA/TA phonons whose group velocities are higher enough and thus successfully tracked. In the case of absorption, electron energy is incremented by that of the absorbed phonon and the phonon energy is deducted from the corresponding spacial bin where the absorption had taken placed. The electron alone is then tracked.
4. Once the electron bundle driven by the external electric field $\vec{F}$ reaches the source terminal, it gets absorbed and the control returns back to step 1. The entire process repeats again by emitting the next electron bundle.

## 4. Results and Discussion

**Figure 2:**
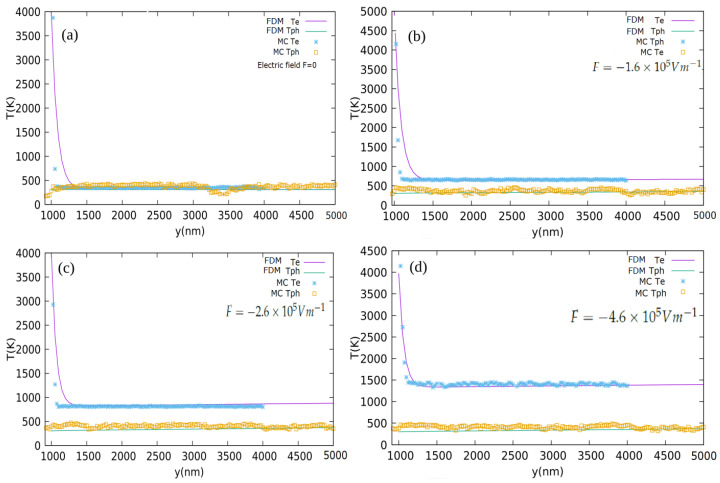
Electron and phonon temperatures ($T_{e}$ and $T_{ph}$) along the *y* direction using MC and the two step model (FDM) for electric fields (**a**) $\vec{F}=0$, (**b**) $\vec{F}=-1.6\times 10^{5}\;Vm^{-1}\hat{y}$, (**c**) $\vec{F}=-2.6\times 10^{5}\;Vm^{-1}\hat{y}$ and (**d**) $\vec{F}=-4.6\times 10^{5}\;Vm^{-1}\hat{y}$.

An electron–phonon MC method is employed to investigate the heat transport in a 2D GaN domain carrying an electron heat source. Both electron as well as phonon temperature in the domain are studied and simulated. Electron temperature in the computational domain is obtained using the relation in Ref. [38]:

(28) $$\frac{3}{2}k_{B}T_{ei}=f\left(\frac{1}{2}m^{*}<v{>}_{i}^{2}-\frac{1}{2}m^{*}v_{d}^{2}\right),$$

where *f*, $m^{*}$, $<v{>}_{i}^{2}$ and $v_{d}$ refer to the electron fraction, effective mass, mean-square velocity, and drift velocity, respectively. Here $T_{ei}$ is the electron temperature for the *i*th bin. A plot of $T_{e}$ along with lattice temperature is shown in Figure 2a–d. As we can see, the temperature of electrons entering from the source is decayed as they drift towards the sink/drain under the electric field $\vec{F}$. The electrons interact with phonons by significantly transferring their energy to them; at the same time, the electric field pumps energy to the electrons. Electron temperature at the source is set to be very high while the lattice temperature is kept at room temperature at both the source and the sink. We remark that the electron temperature defined here is not equal to the true or thermodynamic temperature of the electron ensemble. For electron concentrations in the degenerate range, the temperature defined above exceeds the true temperature of the electron ensemble because the Pauli exclusion principle put limits on the number of particles in the low-energy states, inevitably raising the average energy of the ensemble.

A two temperature model to describe the temperature distribution is proposed. The model is derived from a conventional theory for modeling ultra-fast heating phenomena such as femto/pico second pulsed laser or electron beam induced heating. The most commonly found theory for modeling the ultra-fast heating phenomena is a two step/temperature model that consists of a set of two parabolic heat diffusion equation for electrons and phonons given by [26],

(29) $$\rho_{e}C_{e}\frac{\partial T_{e}}{\partial t}=\kappa_{e}\nabla^{2}T_{e}-G\left(T_{e}-T_{ph}\right)+S,$$

(30) $$\rho_{l}C_{l}\frac{\partial T_{ph}}{\partial t}=\kappa_{l}\nabla^{2}T_{ph}+G\left(T_{e}-T_{ph}\right),$$

where ρ is the electron/phonon density, *C* is the electron/phonon heat capacity, *G* is the electron–phonon coupling constant, κ is the electron/lattice thermal conductivity, *S* is the volumetric heat source term. Through initial intensive heating by a pulsed laser or electron beam, electron temperature rises while lattice temperature remains the same. As time passes, electrons interact with lattice vibrations/phonons, efficiently passing the energy to them. This heats up the lattice and the lattice temperature rises at the cost of decrease in the electron temperature. For *S* = 0, the scenario without a heat source, eventually the lattice temperature catches up to the electron temperature. In many cases the term $\kappa_{l}\nabla^{2}T_{ph}\ll G\left(T_{e}-T_{ph}\right)$; therefore, the above set of equations becomes,

(31) $$\rho_{e}C_{e}\frac{\partial T_{e}}{\partial t}=\kappa_{e}\nabla^{2}T_{e}-G\left(T_{e}-T_{ph}\right)+S,$$

(32) $$\rho_{l}C_{l}\frac{\partial T_{ph}}{\partial t}=G\left(T_{e}-T_{ph}\right).$$

The pulsed laser or electron beam mentioned here is localized in time and the parabolic set equations above are valid for the pulse duration, $t_{pulse}$ greater than the relaxation time of electrons. What we have in the present study is not a pulsed source that is localized in time but a continuous, steady, energetic, electron heat source which is localized in space as we can see in Figure 1. Since the source is not localized in time but in space, Equations (31) and (32) can be modified to get a new model that can take care of the present situation. This is done by exchanging the time and space components and introducing some arbitrary fitting parameters to take care of the dimensionality of the equations. Electrons with high energy and therefore high electron temperature are continuously entering the domain channel from the source terminal. These electrons act as a spatially localized heat source for a given electric field. The two step model formulated to deal with the electron temperature ($T_{e}$) and phonon/lattice temperature ($T_{ph}$) in the channel is given by,

(33) $$Hk_{e}\nabla T_{e}=Ac_{e}\rho_{e}\frac{\partial^{2}T_{e}}{\partial t^{2}}-BG\left(T_{e}-T_{ph}\right)+S,$$

(34) $$k_{l}D\nabla T_{ph}=G\left(T_{e}-T_{ph}\right),$$

where *H* *A*, *B*, *D* are arbitrary fitting parameters. $k_{e}$, $k_{l}$, $c_{e}$, *G*, $\rho_{e}$ and *S* are electron thermal conductivity, lattice thermal conductivity, electron specific heat, the electron–phonon coupling constant, the electron density and the internal heat source term, respectively. Since the electron heat source stretches all the way along the z direction having length $L_{z}$ and thickness $▵L_{y}$, two step model in 1D along the *y* direction is sufficient to address the problem. Hence, the above set of equations in 3D can be reduced into 1D as,

(35) $$Hk_{e}\frac{\partial T_{e}}{\partial y}=Ac_{e}\rho_{e}\frac{\partial^{2}T_{e}}{\partial t^{2}}-BG\left(T_{e}-T_{ph}\right)+S,$$

(36) $$k_{l}D\frac{\partial T_{ph}}{\partial y}=G\left(T_{e}-T_{ph}\right).$$

The above equations in one dimension can be easily solved using the finite difference method (FDM). First, a 2D mesh consisting of space (*y*) and time (*t*) discrete variables is constructed. Second, the set of differential equations are converted to an iterative form where *i* and *j* stand for time and space indices, respectively, see Equations (37) and (38).

(37) $$T_{ph}^{ij}=T_{ph}^{ij-1}+▵yG^{\prime }\left(T_{e}^{ij-1}-T_{ph}^{ij-1}\right),$$

(38) $$T_{e}^{ij}=T_{e}^{ij-1}+A^{\prime }▵y\frac{\left(T_{e}^{i+1j-1}-2T_{e}^{ij-1}+T_{e}^{i-1j-1}\right)}{▵t^{2}}-B^{\prime }▵y\left(T_{e}^{ij-1}-T_{ph}^{ij-1}\right)+C^{\prime }▵yS^{j},$$

where $G^{\prime }$, $A^{\prime }$, $B^{\prime }$ and $C^{\prime }$ absorb all other constants into them. An iterative form of the model is solved after imposing suitable boundary conditions. FDM Equations (37) and (38) solved and plotted along with MC results can be found in Figure 2a–d. When the electrons drift away from the source towards the sink/drain under the influence of the electric field, more and more energy is transferred to the lattice by means of electron–phonon coupling. The stronger the coupling constant *G*, the faster the energy exchange between the electrons and phonons.

**Figure 3:**
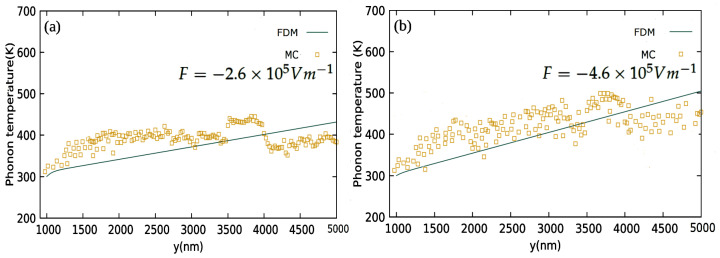
Phonon temperature ($T_{ph}$) along the *y* direction using the MC and two step model (FDM) for uniform electric fields (**a**) $\vec{F}=-2.6\times 10^{5}\;Vm^{-1}\hat{y}$ and (**b**) $\vec{F}=-4.6\times 10^{5}\;Vm^{-1}\hat{y}$.

The categories of EPI scattering that have been studied are electron longitudinal acoustic (LA), electron longitudinal optical (LO), intervalley (IV) and electron polar optical phonon (POP) to characterize the electronic influence of heat transport in GaN. Among them, POP and IV are found to be the dominant players. Moreover, the phonon emission probabilities are much stronger than their absorption counterparts because electron energies are much higher when compared to those of phonons. Therefore, high energy electrons are more likely to lose energy by emitting phonons than to gain energy by absorbing phonons. The electric field serves as a driving force that causes the electrons to drift across the domain from the source to the drain and also pumps energy to the electrons by accelerating them during the flight between two collisions. The energized electrons dissipate energy to the lattice by vigorously interacting with phonons. Hence, the electric field acts as an indirect internal heat source existing throughout the domain. The term “*S*” in Equation (35) represents the volumetric heat source in the two step model. High energy electrons entering the domain cause a very high $T_{e}$ at the source boundary as they travel ahead and interact with the phonons by transferring the energy to them, raising the phonon temperature. The majority of the phonons emitted by the electrons which are of the LO mode eventually decay into acoustic modes. As the distance from the source increases, $T_{e}$ decays rapidly and $T_{ph}$ increases, slowly trying to catch up with $T_{e}$, see Figure 3a,b. In the absence of the internal heat source/electric filed, $T_{ph}$ is found to readily catch up with $T_{e}$, as seen in Figure 2a; in contrast, for non-zero fields, there always exists a gap between $T_{e}$ and $T_{ph}$, i.e., $T_{ph}$ is never able to catch up with $T_{e}$, as seen in Figure 2b–d. With the strength of the electric field increasing, the gap between $T_{e}$ and $T_{ph}$ also widens. Overall, the MC data is found to be in a good agreement with the model.

Next we examined the case of the non-uniform electric field impact on the electron–phonon coupled transport using the two step model and the same is verified using the electron–phonon MC. First, the electric field $\vec{F}\left(y\right)$ is modified into non-uniform form with a constant peak at the $y_{1}<y<y_{2}$ region of the channel. The function $\vec{F}\left(y\right)$ takes the form,

(39) $$\vec{F}\left(y\right)=\left\{\begin{array}{cc} -F_{max}\hat{y}, & \;\mathrm{for}\;y_{1}<y<y_{2},\\ -F_{min}\hat{y}, & \;\mathrm{elsewhere}. \end{array}\right.$$

**Figure 4:**
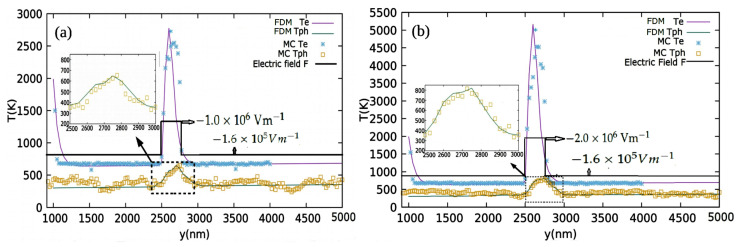
(**a**) Electron and phonon temperatures ($T_{e}$ and $T_{ph}$) along the *y* direction using the MC and the two step model (FDM) for the non-uniform electric field ($\vec{F}=-1.0\times 10^{6}$ Vm${}^{-1}\hat{y}$) peaking at 2500–2800 nm. A sharp peak in $T_{e}$ is observed in the high electric field region and a corresponding rise in $T_{ph}$ is magnified and separately shown as inset. (**b**) Electron and phonon temperatures along the *y* direction using MC and two step model (FDM) for non-uniform electric field ($\vec{F}\left(y\right)=-2.0\times 10^{6}$ Vm${}^{-1}\hat{y}$) peaking at 2500–2800 nm.

$\vec{F}\left(y\right)$, the electron temperature ($T_{e}$) and phonon temperature ($T_{ph}$) are obtained using the MC and the two step model/FDM, a magnified version of $T_{ph}$ in the range $y_{1}<y<y_{2}$ ($y_{1}=2500$ nm, $y_{2}=2800$ nm) for $F_{max}=1.0\times 10^{6}$ Vm${}^{-1}$ and $F_{max}=2.0\times 10^{6}$ Vm${}^{-1}$, shown in Figure 4a,b, respectively. $F_{min}=1.6\times 10^{5}$ Vm${}^{-1}$ is set for both cases.

Second, the volumetric heat source term *S* in the two step model in Equation (35) is also modified in accordance with $\vec{F}\left(y\right)$ to incorporate the non-uniform electric field effect. As we can see, the high energy electrons emitted by the source undergo vigorous interactions with phonons, thus rapidly loosing their energy, raising the phonon energy and phonon temperature. At the same time, the electric field pumps extra energy to the electron as they drift through. The electric field sharply increases at $y=y_{1}$ from $F_{min}$ to $F_{max}$, resulting in the electrons receiving a lot of extra energy and $T_{e}$ rising sharply. The increase in $T_{e}$ also leads to a corresponding increase in $T_{ph}$ due to strong electron–phonon interaction. Since $\vec{F}\left(y\right)$ returns to $F_{min}$ at $y=y_{2}$, both $T_{e}$ and $T_{ph}$ also return back to the original quite abruptly. This creates localized peaks of $T_{e}$ and $T_{ph}$ at $y_{1}<y<y_{2}$. The amplitude of the peak is found to be proportional to the magnitude of $F_{max}$, which is evident in comparing Figure 4a,b. As far as the whole scenario is concerned, the MC simulation and two step model are found to be in good agreement.

## 5. Conclusions

The thermal energy transport in semiconductors is mostly governed by phonons. However, in polar semiconductors like GaN, electronic contribution to the thermal transport is significantly high. In this paper, we used a electron–phonon Monte Carlo to study temperature distribution and thermal properties in a two-dimensional GaN computational domain with a localized, steady, continuous electron heat source. High energy electrons entering the domain from the source and interacting with the phonons drift under the influence ofan external electric field. A two step/temperature analytical model is proposed to describe the electron as well as phonon temperature profiles and is solved using FDM. FDM data is then compared with the MC simulation results. Both FDM and MC simulation data were found to be in a good agreement. The electric field is found to act as an internal volumetric heat source as it continuously pumps energy to the electrons. While the phonon temperature readily catches up with electron temperature under zero field conditions, there always exists a gap between them for non-zero fields. Gap size is found to be directly proportional to the intensity of the electric field. We have also examined the case of non-uniform electric field impact on the electron–phonon coupled transport using the two step model and the same is verified using the electron–phonon MC. Since the computational domain resembles the two dimensional electron gas channel of a typical GaN-based HEMT, this work provides more insight into further investigations on electrothermal properties of HEMTs.

## Data Availability

All data regarding the simulation and modeling are available on request.

## Abbreviations

The following abbreviations are used in this manuscript:

MC	Monte Carlo
BTE	Boltzmann Transport Equation
EPI	Electron Phonon Interaction
FDM	Finite Difference Method
HEMT	High Electron Mobility Transistor
